# Sequential SARS-CoV-2 mRNA Vaccination Induces Anti-Idiotype (Anti-ACE2) Antibodies in K18 Human ACE2 Transgenic Mice

**DOI:** 10.3390/vaccines13030224

**Published:** 2025-02-24

**Authors:** Craig P. Collins, Christian Herzog, Logan V. Vick, Ryan Nielsen, Yanping Izak Harville, Dan L. Longo, John M. Arthur, William J. Murphy

**Affiliations:** 1 School of Medicine, University of California, Davis, CA 95817, USA; cpcollins@ucdavis.edu (C.P.C.); lvvick@ucdavis.edu (L.V.V.); rnnielsen@ucdavis.edu (R.N.); 2 Department of Internal Medicine Nephrology, University of Arkansas for Medical Sciences, Little Rock, AR 72205, USA; herzogchristian@uams.edu (C.H.); yiharville@uams.edu (Y.I.H.); jmarthur@uams.edu (J.M.A.); 3 Department of Medicine, Harvard Medical School, Boston, MA 02115, USA; dlongo@nejm.org; 4 Department of Internal Medicine, Division of Hematology and Oncology, School of Medicine, University of California, Davis, CA 95616, USA

**Keywords:** SARS-CoV-2, vaccination, anti-idiotype antibodies, anti-ACE2 antibodies, K18 mouse model, preclinical SARS-CoV-2 modeling

## Abstract

Background/Objectives: Novel mRNA vaccines have been successfully utilized to curtail the SARS-CoV-2 pandemic. However, the immunology underlying CoV2 vaccinations, particularly with repeated boosting, has not been properly characterized due to limitations in the preclinical modeling of SARS-CoV-2 infection/vaccinations as well as constantly changing vaccine formulations. The immunoregulatory aspects involved in such vaccine approaches remain unclear. Antibodies, due to inherent immunogenicity by VDJ gene rearrangement, have the potential to induce antibodies directed towards them called anti-idiotype antibodies, which can play a downregulatory role in responses. The paratope of some of these anti-idiotype antibodies can also act as a mirror to the original antigen, which, in the case of SARS-CoV-2 vaccines, would be to the spike protein and, therefore, also be capable of binding its target, ACE2, potentially causing adverse effects. Methods: To investigate if sequential SARS-CoV-2 mRNA vaccination can induce anti-idiotype antibody responses, K18 hACE2 transgenic mice were serially vaccinated with a SARS-CoV-2 mRNA construct to determine the kinetics of anti-spike and anti-ACE2 responses via custom-made ELISAs. Results: While sequential vaccination produced robust anti-spike responses, anti-ACE2 levels were also detected and gradually amplified with each boost. These anti-ACE2 antibodies persisted for 3 months after the final vaccination and showed evidence of hACE2 binding, as levels were lower in K18 mice in comparison to the wild type. Conclusions: These data would suggest that sequential SARS-CoV-2 mRNA vaccination has the potential to induce anti-ACE2 antibodies in mice, with each boost amplifying the amount of antibody.

## 1. Introduction

The SARS-CoV-2 pandemic saw the rapid development of protective vaccines, which were widely distributed and successful in curbing the spread of the virus [1]. The most utilized were mRNA vaccines, which encode a modified form of Cov2 spike (S) protein, which cannot fuse to the cell membrane after being cleaved, minimizing the potential for adverse events [2]. The mRNA construct is encapsulated in a lipid nanoparticle delivery system, which prevents degradation of the mRNA, promotes cellular uptake, minimizes TLR triggering, and potentially acts as an adjuvant [3,4]. While other types of vaccines have and still are being used, one consistent requirement of extended protection against Cov2 is the necessity for periodic boosting within a relatively short period of time to amplify the waning antibody response [5]. The continuous emergence of CoV2 variants also necessitates the generation of newer vaccine constructs that adapt the immunogen to the spike protein of the subtype currently causing disease in the community and seasonal boosts [6].

Despite the impressive clinical results, robust immune modeling involving these vaccines has lagged [7,8]. In addition, rare but serious adverse events, such as myocarditis, pulmonary embolism, and thrombosis, have been clinically associated with some vaccines [9]. The modified spike protein, while designed to minimize adverse effects, still has the potential to bind to angiotensin-converting enzyme 2 (ACE2), a protein that plays an important role in regulating blood pressure and inflammation and is expressed in organs such as the lungs, heart, brains, kidneys, and vasculature system [10]. While the benefits of vaccination heavily outweigh the risks posed by SARS-CoV-2 infection, further studies need to be performed to determine the mechanisms underlining these adverse effects.

The SARS-CoV-2 spike protein has issues binding mouse ACE2 (mACE2), causing an obstacle for preclinical modeling [11]. This has led to the usage of Keratin 18 (K18) mice, transgenic mice that express human ACE2 (hACE2) via the cytokeratin 18 promoter [12,13]. While limitations must be taken into consideration with these mice (such as the overexpression of the hACE2 receptor on different cell types and tissue inappropriate expression), they have been utilized for the modeling of SARS-CoV-2 infection and vaccination efficacy [13,14,15,16].

Receptor rearrangement of B cells (for immunoglobulin) and T cells (for the T cell receptor) involves gene rearrangement of the Variable, Diversity, and Joining (VDJ) regions, resulting in a totally new (and foreign) protein based on a lack of thymic selection. Because this is a novel protein, the body has the potential to produce downregulatory antibodies against it in an event known as an “anti-idiotype” response, as originally postulated by the Network Hypothesis by Jerne [17]. Such responses are symmetrical in nature and can be thought of as idiotype–anti-idiotype (Ab1-Ab2), allowing for the immune system to function as a network [18]. Evidence of such immunoregulatory networks has been demonstrated in vivo using hybridoma administration in mouse models and different systems [17,19,20,21,22,23]. These anti-idiotype responses would typically be of a much lower magnitude than the original antibody response [23] but contributed to downregulatory control of the idiotype response [7,24].

Intriguingly, as the anti-idiotype antibody can functionally be a mirror to the idiotype antibody, it could have a similar binding affinity to the idiotype’s target, potentially allowing it to target the receptor that the original antigen targeted [17]. In the scenario of SARS-CoV-2 vaccination or infection, the idiotype antibody would be against the spike protein (anti-spike antibody, or Ab1), and the anti-idiotype antibody would be against the anti-spike protein (anti-anti-spike protein, or Ab2), but due to being a mirror of the idiotype antibody, some paratopes could have the ability to bind the spike protein target receptor, ACE2 (anti-ACE2 antibody) (graphical abstract). This could have biological consequences, with the anti-idiotype antibody having the potential to act as both an agonist or antagonist, to cause receptor internalization, and to mediate antibody-dependent cellular cytotoxicity (ADCC), among other negative outcomes [24]. Anti-ACE2 antibodies have already been noted to develop in some SARS-CoV-2 patients [25,26], having potentially arisen as downregulatory mirror anti-idiotype antibodies. Similarly, the detection of anti-ACE2 antibodies was observed in mice immunized with purified CoV2 S protein [27]. However, the production of these anti-idiotype antibodies in response to vaccination using approved mRNA vaccines has also not been characterized in either mice or humans, though SARS-CoV-2 infection itself in humans has been associated with the development of anti-ACE2 antibodies [25,26,28].

To determine if sequential SARS-CoV-2 vaccination induces anti-idiotype (anti-ACE2) antibody responses, wild type and K18 hACE2 transgenic mice were vaccinated with mRNA-1273 at a dosing schedule comparable to humans. Both wild type and K18 mice produced robust anti-spike antibody responses when vaccinated, which were amplified with each dose. Concurrently, the anti-ACE2 antibody was also detected and was similarly amplified with each dose. These anti-ACE2 antibodies were detectable three months after the final vaccination, while age-matched control mice that were treated with heat-inactivated mRNA-1273 showed no such development. Notably, K18 mice had lower levels of anti-ACE2 after the three-month period, potentially indicating that anti-ACE2 antibodies were binding to the hACE2.

## 2. Materials and Methods

### 2.1. Mice

C57BL/6 and K18 hACE2 transgenic mice were purchased from The UC Davis Mouse Biology Program and monitored for outward abnormalities that would exclude them from the studies, such as the development of lesions or tumors. The mice used in the studies were both male and female, and experiments were started when the mice were between 2 and 3 months old. All mice were group housed (up to 4 mice per cage) in AAALAC-approved specific pathogen-free facilities (The Institute of Regenerative Cures, University of California, Davis) with access to food and water (Teklad 2918 chow) and nesting for enrichment. The mice were housed in microisolator cages and experienced 12 h light/12 h dark cycles. Mice were monitored during all experiments for signs of pathology. All experimental protocols were approved by the IACUC of the University of California, Davis.

### 2.2. Vaccination

Mice were injected subcutaneously in the left flank with 10 µg of expired mRNA-1273 (within 6 months of expiration) in 200 µL of PBS. Control mice were subcutaneously injected with 10 µg of heat-inactivated mRNA-1273 (heated for 20 min at 100 °C) in 200 µL of PBS. Mice were boosted with 10 µg of mRNA-1273 or its heat-inactivated equivalent 7, 28, and 35 days after the initial injection.

### 2.3. Blood Draws

Mice were heated in their cages for approximately 5 min under a heat lamp. Mice were then placed in a holding apparatus, and their tail veins were then nicked with a feather scalpel as superficially as possible. Approximately 100–150 µL of blood was collected per draw. Blood was not collected more than once a week per single mouse. Blood was centrifuged at 1200× *g* for 15 min in acid citrate dextrose tubes to separate out the serum.

### 2.4. ELISA Assays for SARS-CoV-2 and ACE2 Antibodies

A total of 50 µL of a solution of recombinant SARS-CoV-2 receptor-binding domain protein (2 μg/mL, plasmid from BEI (San Francisco, CA, USA)) or recombinant ACE2 protein (2 μg/mL, SinoBiologicals (Wayne, PA, USA), Met1-Ser740 with C-terminal poly His-tail) in a carbonate buffer (0.0125 M Na_2_CO_3_, 0.0875 M NaHCO_3_, pH 9.4) was added to each well of a high-binding ImmunoGrade 96-well plate (MidSci, Fenton, MO, USA) and coated overnight. To determine the presence and concentration of antibodies in plasma or serum, samples were diluted 1:50 in 1% non-fat dry milk PBS-T (1X PBS, 0.1% Tween-20) and added to duplicate wells for 2 h, followed by HRP-conjugated anti-mouse IgG (R&D BioSystems (Minneapolis, MN, USA), HAF007) diluted at 1:5000 in 1% dry milk PBS-T. Before the second antibody and after incubating with the secondary, the plate was washed three times with 250 µL of PBS-t.

**Figure 1:**
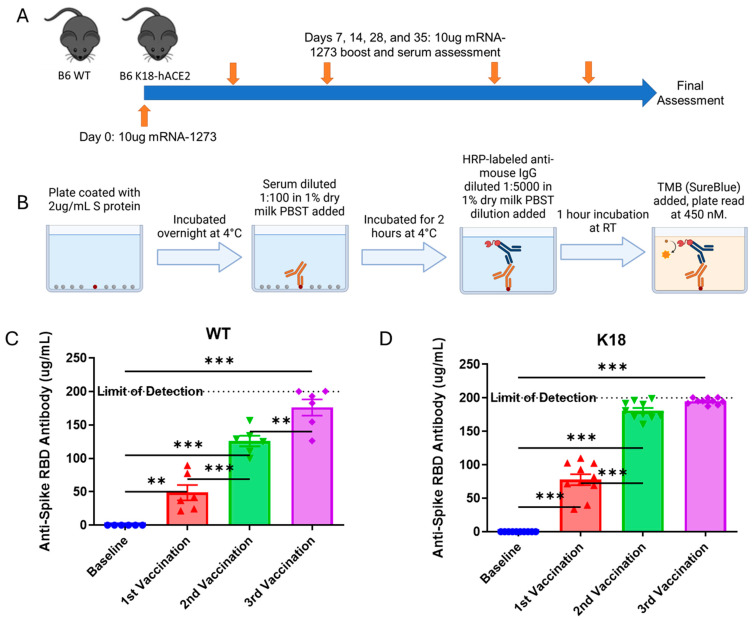
Sequential mRNA-1273 vaccination produces anti-spike antibody responses in wild type and K18 hACE2 transgenic mice. (**A**): Schema. 4–8 week old C57BL/6 and K18 hACE2 transgenic mice were vaccinated subcutaneously with 10ug of mRNA-1273 in 200 uL of PBS, and then boosted on days 7, 14, 28, and 35 with 10ug of mRNA-1273. Mice were assessed for serum anti-spike and anti-ACE2 levels 7 days after each vaccination timepoint. (**B**): Overview of anti-spike antibody ELISA. See methods section for more details. (**C**,**D**): Serum levels of anti-spike RBD antibody in the serum of WT and K18 vaccinated mice over the course of the studies, taken one week after each vaccination timepoint. 1C and 1D: SEM bars, n = 5–10 mice per group, representative of 2 experiments. Mixed-effects ANOVA with multiple comparisons based on means between groups was used to determine statistical significance; *p* < 0.01 **, *p* < 0.001 ***.

**Figure 2:**
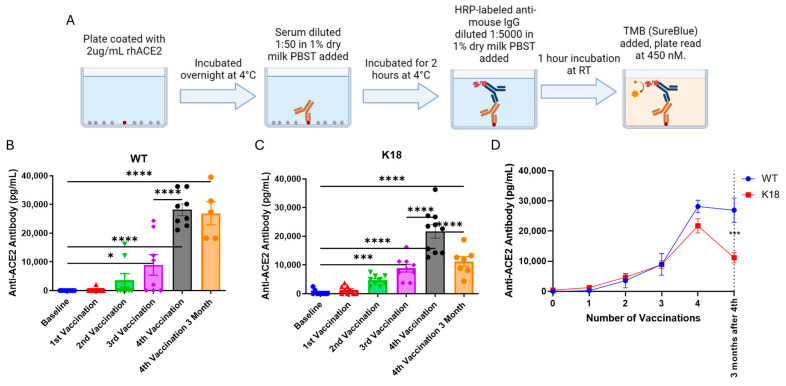
Serial mRNA-1273 vaccination produces gradually amplified anti-ACE2 responses in wild type and K18 hACE2 transgenic mice. (**A**): Overview of anti-ACE2 antibody ELISA. See methods sections for more details. (**B**–**D**): Serum levels of anti-ACE2 antibodies in the serum of WT (**B**) and K18 vaccinated (**C**) mice over the course of the studies (**D**). (**B**,**C**): SEM bars, n = 5–10 mice per group, representative of 2 experiments. Mixed-effects ANOVA with multiple comparisons based on means between groups was used to determine statistical significance; *p* < 0.05 *, *p* < 0.001 ***, *p* < 0.0001 ****. (**D**): Mean and error with standard deviation at each point, n = 5–10 mice per group, representative of 2 experiments. 2-way ANOVA with Tukey’s multiple comparisons test used to determine significance at timepoints; *p* < 0.001 ***.

A total of 75 µL of a solution containing tetramethyl benzidine (SeraCare (Milford, MA, USA)—SureBlue TMB Solution) was added and stopped after 5 min with 75 μL of 1% HCl solution (SeraCare (Mildford, MA, USA)-TMB stop solution). The optical density at 450 nm was determined. The value of a blank well control was subtracted to obtain the final value and reported as OD (450 nm). All measurements were made in duplicate, and the mean value of the two wells was used for the analysis. Recombinant anti-spike (Sinobiologicals (Wayne, PA, USA), Arg319-Phe541 with C-terminal poly His-tail) or anti-ACE2 (Invitrogen, MA5-31395) were used at known concentrations to create a standard curve for the assay (and for a positive control), which was then used to extrapolate the concentration of protein from OD values of test samples. The upper range was 200 µg/mL, as designated on the relevant graphs as the limit of detection in Figure 1 and Figure 2.

A mouse monoclonal anti-human ACE2 antibody (Invitrogen (Carlsbad, CA, USA), MA5-31395) was used as a positive control for the anti-ACE2.

### 2.5. Quantitative Real-Time PCR

Tissue from the site of injection was processed via Qiagen’s (Germantown, MD, USA) RNeasy Fibrous Tissue Mini Kit (74794), and the resulting RNA was quantified via NanoDrop. qPCR was performed using AB Step-ONE Plus (Applied Biosystems, Foster City, CA, USA) in the presence of SYBR Green Supermix (Applied Biosystems). Primer assay for the Spike gene was purchased from Thermo Fisher Scientific (Cat# A50137). mRNA levels were calculated using the comparative threshold cycle method (C_t_). C_t_ values for the housekeeping gene (GAPDH) and for the genes of interest were determined, and the difference between the C_t_ values of each gene of interest and the mean GAPDH Ct was calculated (ΔC_t_). Differences in ΔC_t_ (ΔΔC_t_) of genes of interest in the vaccinated group were normalized to control groups (heat-inactivated vaccinated mice) as shown in the following equation: ΔΔC_t_ = ΔC_t_(sample) − ΔC_t_(mean of control group). RT-PCR data are presented as fold change expression = 2^−ΔΔCt^ in comparison to the control group.

### 2.6. Statistics

Statistical analysis was performed using GraphPad Prism v6.02 (GraphPad Software Inc., La Jolla, CA, USA). Data were expressed as mean ± standard error of the mean (SEM). A non-parametric Mann–Whitney t-test was used to compare two unpaired groups. For the analysis of three or more groups, a mixed-effects ANOVA was utilized. An adjusted *p* value of <0.05 was considered significant. All graphs/data shown are representative of at least two experiments, as further specified in figure legends. Mouse numbers per group and per experiment/time point were between 1 and 10, as further specified in figure legends and demonstrated in individual boxplot points.

### 2.7. Study Approval

All animal studies and protocols were approved by the UC Davis IACUC (protocol numbers 18940 and 20707). Studies were approved by UC Davis biohazard use authorization (BUA) number R1592.

## 3. Results

### 3.1. Sequential mRNA-1273 Vaccination Produces Anti-Spike Antibody Responses in Wild Type and K18 hACE2 Transgenic Mice

Sequential mRNA-1273 vaccination produces gradually amplified anti-spike antibody responses in humans [29,30]. It was first determined if wild type and K18 mice also had this gradual amplification of anti-spike antibody in response to serial vaccination. Wild type and K18 mice were vaccinated with 10 µg of mRNA-1273 subcutaneously and boosted with 10 µg at days 7, 14, 28, and 35 (Figure 1A). A custom-designed ELISA was used to determine the anti-spike antibody presence in the serum of vaccinated mice (Figure 1B).

Sequential vaccination induced robust anti-spike antibody responses in both wild type and K18 mice (Figure 1C,D). These anti-spike responses did not occur in mice that received heat-inactivated mRNA-1273 (Figure S1A). PCR confirmed that the vaccine was incorporated at the injection sites of the mice, with transcription of spike protein being determined to occur; though, as anticipated, transcription itself did not amplify with each boost (Figure S1B). These data suggest that WT and K18 mice mirror human anti-spike response kinetics when serially vaccinated, showing a gradual amplification of anti-spike antibodies with each boost.

### 3.2. Serial mRNA-1273 Vaccination Produces Gradually Amplified Anti-ACE2 Responses in Wild Type and K18 hACE2 Transgenic Mice

The development of anti-ACE2 (anti-idiotype) antibodies was next assessed. A custom ELISA was developed to assess anti-ACE2 antibodies in the serum of vaccinated mice at the same time points assessed previously (Figure 1A and Figure 2A). Anti-ACE2 antibodies could be detected as soon as a week after the initial vaccination in both WT and K18 mice (Figure 2B,C). Further vaccination amplified the amount of the anti-ACE2 antibody observed, peaking a week after the fourth dose of vaccine in both groups of mice. Assessment three months after the fourth dose of vaccine revealed that while levels of anti-ACE2 had decreased, anti-ACE2 was still detectable at levels higher than with one or two vaccinations. K18 mice had lower levels of the anti-ACE2 antibody at this time point when compared to WT mice, suggesting that the antibodies were potentially binding to hACE2 in the tissues of the mice (Figure 2D). It should be noted that levels of anti-ACE2 detected were, proportionally, much less than anti-spike antibody levels at all vaccination points. Mice that received heat-inactivated mRNA-1273 did not form anti-ACE2 antibodies (Figure S2A). These data confirm the development of anti-ACE2 antibodies to mRNA-1273 vaccination and suggest potential binding to hACE2.

## 4. Discussion

The data shown in this study indicate that serial SARS-CoV-2 mRNA-1273 vaccination, while inducing robust anti-spike antibody responses, also promotes the development and gradual amplification of anti-ACE2 antibodies in both WT and K18 mice. It is important to note that the anti-idiotype response is polyclonal in nature, and anti-ACE2 antibodies only constitute a subset of the total anti-idiotype population. More work is needed in characterizing the total anti-idiotype response and effects of continuous antigen exposure (by both the virus and vaccines). These mirror anti-idiotype antibodies were detectable 3 months after the final vaccination, though lower levels were detected in K18 mice, suggesting the potential of receptor binding to hACE2. Further studies would be needed to confirm this binding and the biological effects incurred from the binding, however.

These data must be contextualized by the limitations of the K18 mouse model. K18 mice, due to hACE2 expression being tied to the cytokeratin 18 promoter, have an overexpression of hACE2, as well as expression in cells or tissues that do not align with humans [13,14,15,16]. However, as anti-ACE2 antibodies developed in both WT and K18 mice, this would suggest that the development of these anti-idiotypic antibodies was not tied to the hACE2 expressed by the K18 mice. While data exist showing the development of anti-ACE2 antibodies with SARS-CoV-2 human infection [25,26,28] and vaccination [31,32], these studies have not been correlated with pathological outcomes, requiring more studies to determine these effects.

Others have demonstrated potential effects of anti-ACE2 autoantibody binding, such as complement activation [26] and pro-inflammatory cytokine production [33]. The suppression of anti-ACE2 to counteract these effects, potentially in the form of monoclonal antibodies or convalescent serum to target and bind the antibodies, while keeping the benefits of vaccination, could be investigated with the K18 model, as hACE2 binding differentials with and without treatment could determine biological outcomes. The overexpression of hACE2 must be taken into consideration, however, as this could elevate pathology above that which would be demonstrated in humans, as observed with SARS-CoV-2 infection being much more severe in K18 mice [14].

While heat-inactivated mRNA-1273 was used as a control for our studies (while also using it to rule out unexpected anti-spike or anti-ACE2 antibody development to the LNP carrier itself), future studies could benefit from looking at more direct effects of the antibodies induced from vaccination. Anti-spike antibodies could be purified and used to vaccinate mice to determine if they have the direct ability to induce anti-ACE2 antibody development, independent of the mRNA construct or LNP vehicle. Conversely, anti-ACE2 could be investigated to determine potential biological effects, which would be particularly relevant given the heterologous human population, by which factors like age, sex, and obesity have a notable impact on the expression of ACE2 [34,35,36]—a dose–response of anti-ACE2 could be revealing. Determining levels of anti-ACE2 in human patients after vaccination and correlating with adverse effects would also be recommended. A partial blockade of ACE2 in mice to mimic the downregulation of ACE2, which has been associated with more severe SARS-CoV-2 pathology [35,36], could also be implemented, as ACE2 expression levels have not been properly investigated in the context of vaccination adverse events. Anti-idiotype antibodies to other SARS-CoV-2 targets, like NRP-1, should also be investigated, especially regarding potential PASC symptom development. Given how little focus anti-ACE2 antibodies have been given, even after having been shown to develop with correlations to SARS-CoV-2 severity being made [26,28], there are many potential paths to explore with both infection and vaccination in mice as well as interventions, and our studies represent only the beginning of understanding this phenomenon.

Determining the degree of anti-ACE2 development in K18 mice when challenged with SARS-CoV-2 is something that must be investigated as well and potentially compared to levels observed vs. vaccination. Determining the amount of anti-ACE2 to have biological effects would also be prudent, though it should be kept in mind that viral infection has been demonstrated in humans to induce higher amounts of anti-ACE2 antibodies than vaccinations [33] and thus would presumably have a higher chance of incurring anti-idiotype-related effects. The use of mouse-adapted SARS-CoV-2 could also be highly beneficial for understanding anti-idiotype antibody development, but adverse effects from anti-ACE2 binding could be missed if WT mice are used in modeling.

With variants of SARS-CoV-2 arising at a rapid rate, different formulations of the mRNA vaccines have been employed and are now recommended as seasonal boosters. However, we chose to use the original vaccine formulation throughout this manuscript. This is due to the majority of the population having received this original formulation. It would be anticipated that these newer formulations, due to having similar constructs and delivery vehicles, would have the same potential of inducing anti-ACE2 antibodies if given at the same dosing schedule, or even with seasonal boosting, as they are now being used. We do acknowledge that measuring 3 months after the final boost of the vaccine in our model was not sufficient in showing the true long-term persistence potential of the anti-ACE2 antibodies and that boosting after a prolonged period of time to mimic seasonal boosting was also not incorporated in our modeling. We also acknowledge that only using one formulation of mRNA vaccine (mRNA-1273) could lead to a formula-specific abnormality being misattributed to other formulations, and the vaccination regimen used in our studies was more condensed than clinically applied. However, another caveat is that mice, unlike humans, are not continuously exposed to potential CoV2 infections, which may further amplify such anti-idiotype responses. Mouse-to-human age equivalencies must also be taken into consideration when designing the boosting schedule, as weeks for mice can correspond to years in humans [37].

## 5. Conclusions

In conclusion, our studies demonstrate that serial SARS-CoV-2 mRNA vaccination has the potential to induce anti-idiotypic antibody responses capable of binding hACE2. It will be of interest to further assess these responses using other CoV2 vaccine formulations as well as assessing in mice with human-modifying variables, such as aging and obesity.

## Data Availability

The raw data can be found as an Excel sheet in the Supplemental Materials.

## Supplementary Materials

The following supporting information can be downloaded at https://www.mdpi.com/article/10.3390/vaccines13030224/s1, Figure S1: Heat inactivated vaccination produces no anti-spike antibody response in WT or K18 mice; Figure S2: Heat inactivated vaccination produces no anti-ACE2 antibody response in WT or K18 mice.

## References

[1] Chong S.H., Burn L.A., Cheng T.K.M., Warr I.S., Kenyon J.C. (2022). A Review of COVID Vaccines: Success against a Moving Target. Br. Med. Bull..

[2] Li C., Lee A., Grigoryan L., Arunachalam P.S., Scott M.K.D., Trisal M., Wimmers F., Sanyal M., Weidenbacher P.A., Feng Y. (2022). Mechanisms of Innate and Adaptive Immunity to the Pfizer-BioNTech BNT162b2 Vaccine. Nat. Immunol..

[3] Alameh M.-G., Tombácz I., Bettini E., Lederer K., Sittplangkoon C., Wilmore J.R., Gaudette B.T., Soliman O.Y., Pine M., Hicks P. (2021). Lipid Nanoparticles Enhance the Efficacy of MRNA and Protein Subunit Vaccines by Inducing Robust T Follicular Helper Cell and Humoral Responses. Immunity.

[4] Hou X., Zaks T., Langer R., Dong Y. (2021). Lipid Nanoparticles for MRNA Delivery. Nat. Rev. Mater..

[5] Dogra P., Schiavone C., Wang Z., Ruiz-Ramírez J., Caserta S., Staquicini D.I., Markosian C., Wang J., Sostman H.D., Pasqualini R. (2023). A Modeling-Based Approach to Optimize COVID-19 Vaccine Dosing Schedules for Improved Protection. JCI Insight.

[6] Tao K., Tzou P.L., Nouhin J., Gupta R.K., de Oliveira T., Kosakovsky Pond S.L., Fera D., Shafer R.W. (2021). The Biological and Clinical Significance of Emerging SARS-CoV-2 Variants. Nat. Rev. Genet..

[7] Collins C.P., Longo D.L., Murphy W.J. (2024). The Immunobiology of SARS-CoV-2 Infection and Vaccine Responses: Potential Influences of Cross-Reactive Memory Responses and Aging on Efficacy and off-Target Effects. Front. Immunol..

[8] Lipworth W., Kerridge I., Stewart C., Silva D., Upshur R. (2023). The Fragility of Scientific Rigour and Integrity in “Sped up Science”: Research Misconduct, Bias, and Hype and in the COVID-19 Pandemic. J. Bioeth. Inq..

[9] Klein N.P., Lewis N., Goddard K., Fireman B., Zerbo O., Hanson K.E., Donahue J.G., Kharbanda E.O., Naleway A., Nelson J.C. (2021). Surveillance for Adverse Events After COVID-19 MRNA Vaccination. JAMA.

[10] Patel V.B., Zhong J.-C., Grant M.B., Oudit G.Y. (2016). Role of the ACE2/Angiotensin 1–7 Axis of the Renin–Angiotensin System in Heart Failure. Circ. Res..

[11] Shang J., Ye G., Shi K., Wan Y., Luo C., Aihara H., Geng Q., Auerbach A., Li F. (2020). Structural Basis of Receptor Recognition by SARS-CoV-2. Nature.

[12] Sun J., Zhuang Z., Zheng J., Li K., Wong R.L.-Y., Liu D., Huang J., He J., Zhu A., Zhao J. (2020). Generation of a Broadly Useful Model for COVID-19 Pathogenesis, Vaccination, and Treatment. Cell.

[13] McCray P.B., Pewe L., Wohlford-Lenane C., Hickey M., Manzel L., Shi L., Netland J., Jia H.P., Halabi C., Sigmund C.D. (2007). Lethal Infection of K18-HACE2 Mice Infected with Severe Acute Respiratory Syndrome Coronavirus. J. Virol..

[14] Moreau G.B., Burgess S.L., Sturek J.M., Donlan A.N., Petri W.A., Mann B.J. (2020). Evaluation of K18-HACE2 Mice as a Model of SARS-CoV-2 Infection. Am. J. Trop. Med. Hyg..

[15] Oladunni F.S., Park J.-G., Pino P.A., Gonzalez O., Akhter A., Allué-Guardia A., Olmo-Fontánez A., Gautam S., Garcia-Vilanova A., Ye C. (2020). Lethality of SARS-CoV-2 Infection in K18 Human Angiotensin-Converting Enzyme 2 Transgenic Mice. Nat. Commun..

[16] Winkler E.S., Chen R.E., Alam F., Yildiz S., Case J.B., Uccellini M.B., Holtzman M.J., Garcia-Sastre A., Schotsaert M., Diamond M.S. (2022). SARS-CoV-2 Causes Lung Infection without Severe Disease in Human ACE2 Knock-In Mice. J. Virol..

[17] Jerne N.K. (1974). Towards a Network Theory of the Immune System. Ann. Immunol..

[18] Köhler H. (1975). The Response to Phosphorylcholine: Dissecting an Immune Response. Transplant. Rev..

[19] Kearney J.F., Barletta R., Quan Z.S., Quintáns J. (1981). Monoclonal vs. Heterogeneous Anti-H-8 Antibodies in the Analysis of the Anti-Phosphorylcholine Response in BALB/c Mice. Eur. J. Immunol..

[20] Wittner M.K., Bach M.A., Köhler H. (1982). Immune Response to Phosphorylcholine. IX. Characterization of Hybridoma Anti-TEPC15 Antibodies. J. Immunol..

[21] Cosenza H. (1976). Detection of Anti-Idiotype Reactive Cells in the Response to Phosphorylcholine. Eur. J. Immunol..

[22] Cosenza H., Köhler H. (1972). Specific Suppression of the Antibody Response by Antibodies to Receptors. Proc. Natl. Acad. Sci. USA.

[23] Kluskens L., Köhler H. (1974). Regulation of Immune Response by Autogenous Antibody against Receptor. Proc. Natl. Acad. Sci. USA.

[24] Murphy W.J., Longo D.L. (2021). A Possible Role for Anti-Idiotype Antibodies in SARS-CoV-2 Infection and Vaccination. N. Engl. J. Med..

[25] Arthur J.M., Forrest J.C., Boehme K.W., Kennedy J.L., Owens S., Herzog C., Liu J., Harville T.O. (2021). Development of ACE2 Autoantibodies after SARS-CoV-2 Infection. PLoS ONE.

[26] Casciola-Rosen L., Thiemann D.R., Andrade F., Trejo-Zambrano M.I., Leonard E.K., Spangler J.B., Skinner N.E., Bailey J., Yegnasubramanian S., Wang R. (2022). IgM Anti-ACE2 Autoantibodies in Severe COVID-19 Activate Complement and Perturb Vascular Endothelial Function. JCI Insight.

[27] Lai Y.-C., Cheng Y.-W., Chao C.-H., Chang Y.-Y., Chen C.-D., Tsai W.-J., Wang S., Lin Y.-S., Chang C.-P., Chuang W.-J. (2022). Antigenic Cross-Reactivity Between SARS-CoV-2 S1-RBD and Its Receptor ACE2. Front. Immunol..

[28] Hallmann E., Sikora D., Poniedziałek B., Szymański K., Kondratiuk K., Żurawski J., Brydak L., Rzymski P. (2023). IgG Autoantibodies against ACE2 in SARS-CoV-2 Infected Patients. J. Med. Virol..

[29] Choi A., Koch M., Wu K., Chu L., Ma L., Hill A., Nunna N., Huang W., Oestreicher J., Colpitts T. (2021). Safety and Immunogenicity of SARS-CoV-2 Variant MRNA Vaccine Boosters in Healthy Adults: An Interim Analysis. Nat. Med..

[30] Bag Soytas R., Cengiz M., Islamoglu M.S., Borku Uysal B., Yavuzer S., Yavuzer H. (2022). Antibody Responses to COVID-19 Vaccines in Older Adults. J. Med. Virol..

[31] Bellucci M., Bozzano F.M., Castellano C., Pesce G., Beronio A., Farshchi A.H., Limongelli A., Uccelli A., Benedetti L., De Maria A. (2024). Post-SARS-CoV-2 Infection and Post-Vaccine-Related Neurological Complications Share Clinical Features and the Same Positivity to Anti-ACE2 Antibodies. Front. Immunol..

[32] Tsoi J.Y.H., Cai J., Situ J., Lam W.J., Shun E.H.K., Leung J.K.Y., Chen L.L., Chan B.P.C., Yeung M.L., Li X. (2023). Autoantibodies against Angiotensin-Converting Enzyme 2 (ACE2) after COVID-19 Infection or Vaccination. J. Med. Virol..

[33] Geanes E.S., McLennan R., LeMaster C., Bradley T. (2024). Autoantibodies to ACE2 and Immune Molecules Are Associated with COVID-19 Disease Severity. Commun. Med..

[34] Chen J., Jiang Q., Xia X., Liu K., Yu Z., Tao W., Gong W., Han J.-D.J. (2020). Individual Variation of the SARS-CoV-2 Receptor ACE2 Gene Expression and Regulation. Aging Cell.

[35] Rodrigues R., Costa de Oliveira S. (2021). The Impact of Angiotensin-Converting Enzyme 2 (ACE2) Expression Levels in Patients with Comorbidities on COVID-19 Severity: A Comprehensive Review. Microorganisms.

[36] Tavares C.d.A.M., Avelino-Silva T.J., Benard G., Cardozo F.A.M., Fernandes J.R., Girardi A.C.C., Jacob Filho W. (2020). ACE2 Expression and Risk Factors for COVID-19 Severity in Patients with Advanced Age. Arq. Bras. Cardiol..

[37] When Are Mice Considered Old?. https://www.jax.org/news-and-insights/jax-blog/2017/november/when-are-mice-considered-old.

