# Supplementary material for: Sequential SARS-CoV-2 mRNA Vaccination Induces Anti-Idiotype (Anti-ACE2) Antibodies in K18 Human ACE2 Transgenic Mice
Source: Vaccines (Basel). 2025 Feb 24;13(3):224. doi: 10.3390/vaccines13030224 (PMC11946769; doi:10.3390/vaccines13030224)
Supplement: Supplementary file 1 [file vaccines-13-00224-s001.zip › vaccines-3413051-supplementary.pdf]

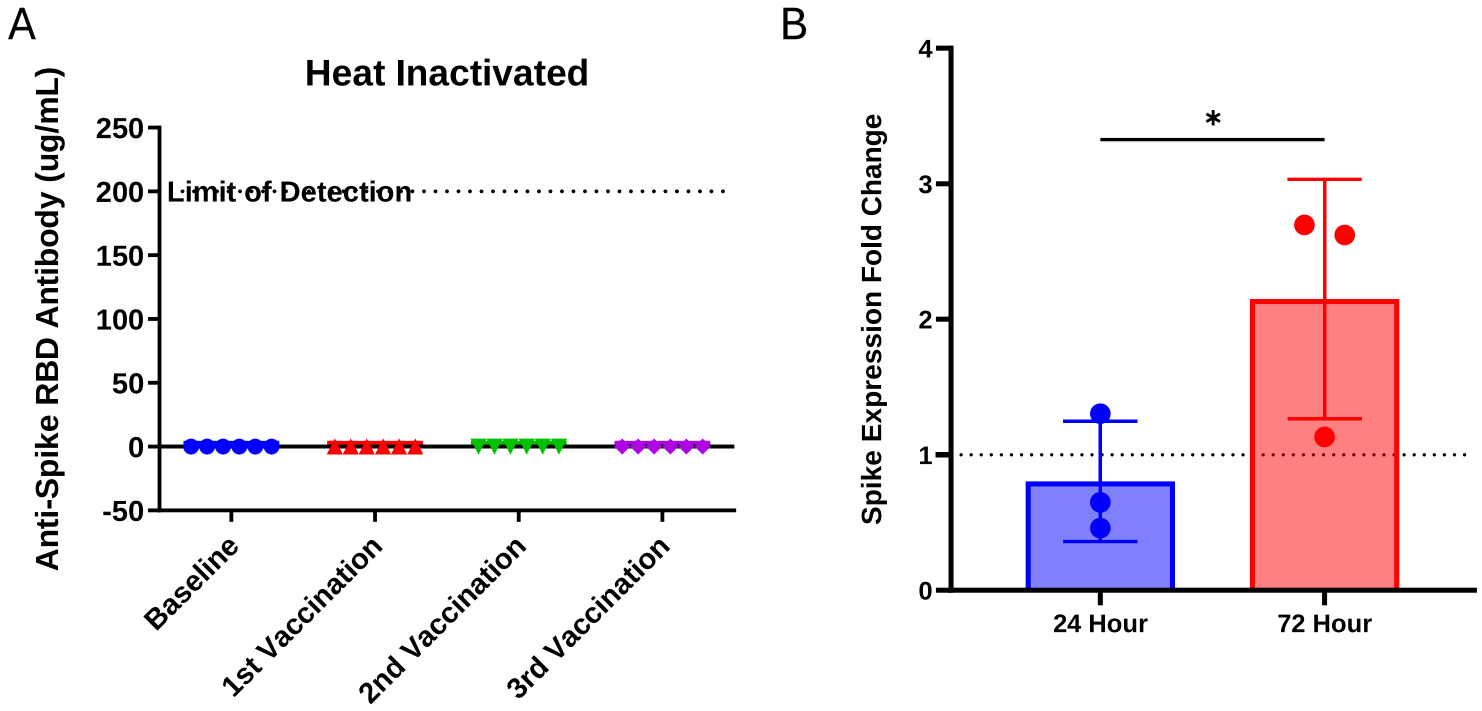

Supplemental Figure 1: Heat inactivated vaccination produces no anti-spike antibody response in WT or K18 mice. **(S1A):** Serum levels of anti-spike RBD antibody in the serum of WT and K18 heat inactivated vaccinated mice over the course of the studies. (**S1B**)**:** PCR of spike transcription at the injection site of vaccinated WT mice 24 and 72 hours after the first vaccination **S1A:** SEM bars, n=6 mice per group, representative of 2 experiments. Mixed-effects ANOVA with multiple comparisons based on means between groups was used to determine lack of statistical significance at $P>0.05$. **S1B** N=3 mice per group, representative of 1 experiment. One Tailed Student T test was used to determine statistical significance; $P<0.05$*.

A

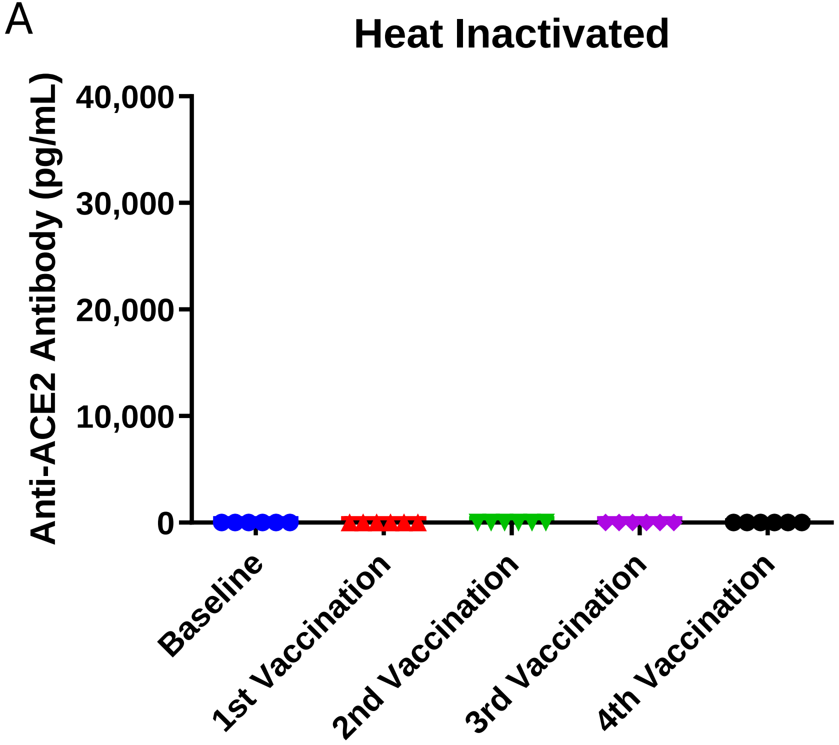

Supplemental Figure 2: Heat inactivated vaccination produces no anti-ACE2 antibody response in WT or K18 mice. **(S2A):** Serum levels of anti-ACE2 antibody in the serum of WT and K18 heat inactivated vaccinated mice over the course of the studies. **S2A:** SEM bars, n=6 mice per group, representative of 2 experiments. Mixed-effects ANOVA with multiple comparisons based on means between groups was used to determine lack of statistical significance at P>0.05.
